# The recovery and independence of elbow flexion and forearm supination after Oberlin II transfer in brachial plexus injuries: a long term follows up study

**DOI:** 10.1007/s12306-024-00863-9

**Published:** 2024-08-30

**Authors:** A. M. Acharya, Nikhil Hegde, Anil K. Bhat

**Affiliations:** 1 Department of Hand Surgery, Kasturba Medical College, Manipal, Manipal Academy of Higher Education, Manipal, 576104 India; 2 Consultant Orthopaedic Surgeon, Jayadev Memorial Rastrotthana Hospital and Research Centre, Rajarajeshwari Nagar, BEML 5Th Stage, Bengaluru, 560098 India

**Keywords:** Brachial plexus injury, Musculocutaneous nerve, Nerve transfer, Partial ulnar and median nerve transfer, Oberlin transfer, Supination

## Abstract

**Purpose:**

The Oberlin II double fascicular nerve transfer has been evaluated extensively for objective outcomes for elbow flexion in brachial plexus injuries (BPI). However, there is limited information available on the recovery pattern of supination and patient-reported activity in the long-term. Our study aimed to assess the functional results with a minimum of five years of follow-up.

**Methods:**

We evaluated patients with a minimum of five years after the Oberlin II procedure for post-traumatic BPI. They were evaluated using MRC grading, range of active movements, QuickDASH score and activity to check elbow flexion and forearm supination independent of finger and wrist flexion.

**Results:**

18 out of 26 patients responded with a mean follow-up of 79.4 months (range: 61–98). 16 (88.9%) (p < 0.000) patients recovered to achieve active elbow flexion and forearm supination of either MRC grade 3 power or more. The average range of active elbow flexion was 113.9^°^ (range: 0–140^°^) and active supination was 67.8^°^ (0–90^°^). Patients who achieved grade 3 flexion or higher were found to regain supination after a delay. The recovery continues even after two years of surgery. The mean QuickDASH score was 21.8 (range: 2.3–63.6). There’s a significant inverse correlation between QuickDASH with both flexion and supination (p < .001 and < 0.05). 15 patients (83.3%) could demonstrate a dissociation of elbow and forearm movements from digital and wrist movements.

**Conclusion:**

Our study demonstrated reliable functional results with independent elbow flexion, forearm supination and acceptable patient-reported outcomes for Oberlin II procedure in BPI.

## Introduction

In 1994 Oberlin introduced the novel nerve transfer of a fascicle from the ulnar nerve to the biceps branch of the musculocutaneous nerve (MCN) which revolutionized the treatment of debilitating brachial plexus injuries [1]. Subsequently, Mackinnon et al. and Liverneaux et al. described simultaneously the addition of median nerve fascicle transfer to the brachialis branch of MCN as they believed brachialis to be stronger among the elbow flexors [2, 3]. This was referred to as the Oberlin II procedure [3]. Since then this highly efficient nerve transfer has stood the test of time in the last two decades with gratifying results [4–6]. Although there are reports on long-term results of the Oberlin procedure, there is a lack of information on the Oberlin II transfers [7–10]. This procedure has been evaluated extensively for elbow flexion which is one of the actions of the biceps. However, the prime function of the biceps is supination of the forearm which has not yet been thoroughly evaluated for Oberlin II procedure. [11, 12]. Supination is a very important action required for our activities of daily living like the hand to mouth for eating, buttoning of shirt and ablution for toilet hygiene. Carlsen et al. did report on supination strength using a laboratory instrument, however this method is still not universally standardized and is expensive [12]. A drawback of this procedure despite a powerful generation of elbow flexion is the presence of co-contractures of elbow flexion with the finger and wrist flexors [13]. In many patients, this persists and interferes with activities of daily living since the donor is not synergistic [13]. Although evidence on this aspect has been shown using clinical studies and electromyography, the influence of this effect on the overall function has not been reported in the long term [10, 14, 15]. In this regard, we decided to seek answers on the recovery pattern and the results of Oberlin II transfer in adult brachial plexus injuries with a minimum of five years of follow-up. The aim was to objectively evaluate the power and active range of movement for the elbow and forearm with emphasis on supination along with a patient reported outcome measure (PROM) by means of QuickDASH score.

## Materials and methods

We assessed the case records of 162 patients undergoing surgery for brachial plexus injuries in the Department of Orthopaedics after approval of the institute ethics committee from January 2010 till January 2024. We identified 97 patients who had completed a minimum of five years follow-up among which 28 had suffered brachial plexus injuries involving the axis of either C5, C6 or C5–C7 roots. They underwent either Oberlin I or II transfer for elbow function apart from other nerve procedures. 26 of them had undergone the Oberlin II procedure and were recalled for assessment. We included patients in the age group of 18–50 years and excluded those with fractures of the forearm, elbow and arm that could potentially alter the outcome of Oberlin’s procedure. 18 (69.2%) of the 26 patients responded to return for a follow-up visit to measure outcomes. The Outcomes were assessed using the Medical Research Council (MRC) power grading [16, 17]. The range of forearm and elbow movements was assessed using a pen and a goniometer by a standard technique [18, 19]. The QuickDASH score was used to assess patient-reported functional outcomes as it has been validated and used in the South Asian population and brachial plexus injuries [7, 20]. We also assessed two important functions in patients: 1) Ability to eat with hand without aid or instrument and, 2) Demonstrate independent finger and wrist flexion and extension along with pronation and supination of the forearm while steadily holding the elbow in 90^°^ flexion. The sample size of 18 patient provided adequate power to check differences of ≥ 20% with p ≤ 0.05 between the various groups. Jamovi software was used to perform all the statistical analyses. Entry of data was based on mean ± SD. The Mann–Whitney U test, was used to compare the distributions of independent groups. Pearson’s correlation coefficient and Spearman’s correlation coefficient were used as measures of the strength and direction of the relationship between variables. Pearson’s chi-square test was used to determine significant associations between categorical variables.

### Surgical procedure

All patients underwent routine supra-clavicular brachial plexus exploration. In C5, C6 or C5–7 level injuries we routinely planned nerve reconstruction for the stability of the shoulder, elbow and pain relief.This involved nerve grafting after freshening the native proximal neuroma. If found viable, they were bridged to the upper and middle trunk or branches. In addition, nerve transfers were done depending on the availability of viable donors for 1) Suprascapular and axillary nerve, 2) Oberlin II transfer for MCN, 3) Supraclavicular nerve transfer to upper trunk branches for sensation and pain relief. Nerve transfers were the only option when proximal roots were avulsed or had doubtful viability.

### Oberlin II procedure

**Fig. 1:**
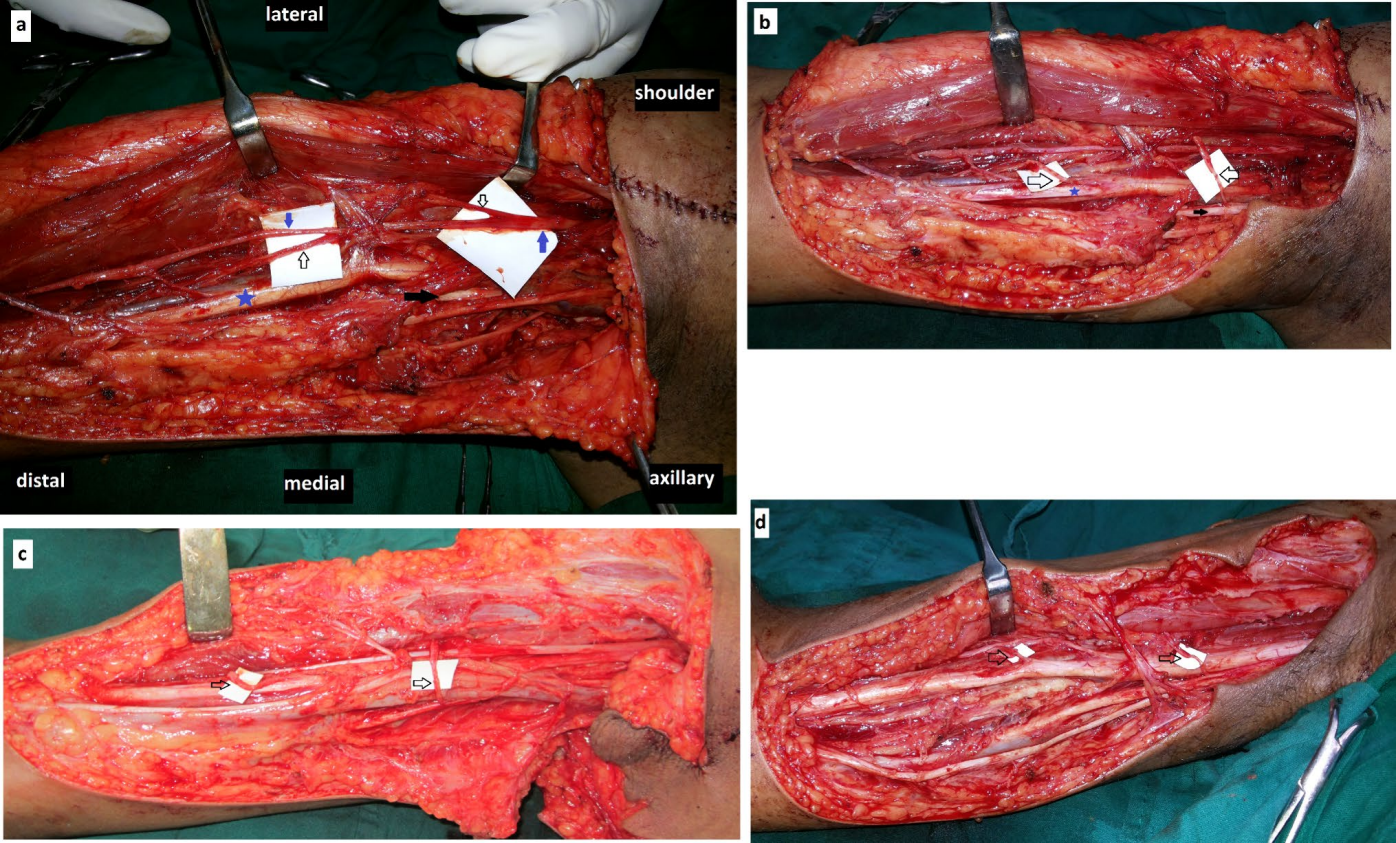
**a** Showing the course of the musculocutaneous (blue arrow pointing up), median (blue star) and ulnar nerve (solid black arrow). Notice the course of biceps branch (black arrow pointing down), brachialis branch (black arrow pointing down) and the terminal lateral cutaneous nerve of forearm (blue arrow pointing down). **b** Notice the Oberlin II procedure with ulnar nerve (solid black arrow) fascicle being transferred and coapted (black arrow pointing distally) to the biceps branch of the musculocutaneous nerve. A fascicle from median nerve has been transferred (black arrow pointing proximally) to the brachialis branch. **c**, **d** Notice the relative proximity of the median nerve fascicle transfer (black arrow) towards the targeted brachialis muscle that the ulnar nerve fascicle towards biceps muscle in two other patients which is similar to **b**

The patient is positioned supine with the arm abducted at 90^°^. A zig-zag incision is made at the axilla and continued anteromedially till the distal fourth of the arm. A plane is developed between the biceps and coracobrachialis and the MCN is identified. It is dissected distally to identify the biceps, brachialis and the terminal sensory branches which are observed in the proximal 1/3rd and distal 1/3rd of the arm respectively [2, 3]. The ulnar and median nerve is identified and after an epineurotomy, a fascicle matching the size of the respective biceps and brachialis branch of MCN is harvested [2, 3] (Fig. 1). In case of median nerve, an anterior fascicle, and for ulnar nerve, an anterolateral fascicle is selected [21, 22]. The ulnar nerve fascicle was transferred to the biceps branch and the median nerve fascicle to the brachialis branch [2, 3] (Fig. 1). In cases of C5-7 palsy, we have used a nerve stimulator to confirm the activity of the fascicle from the median nerve [2, 3, 23, ]. All the patients were immobilized in a shoulder and elbow brace for four weeks and subsequently started on an elbow and hand therapy program.

## Results

**Fig. 2:**
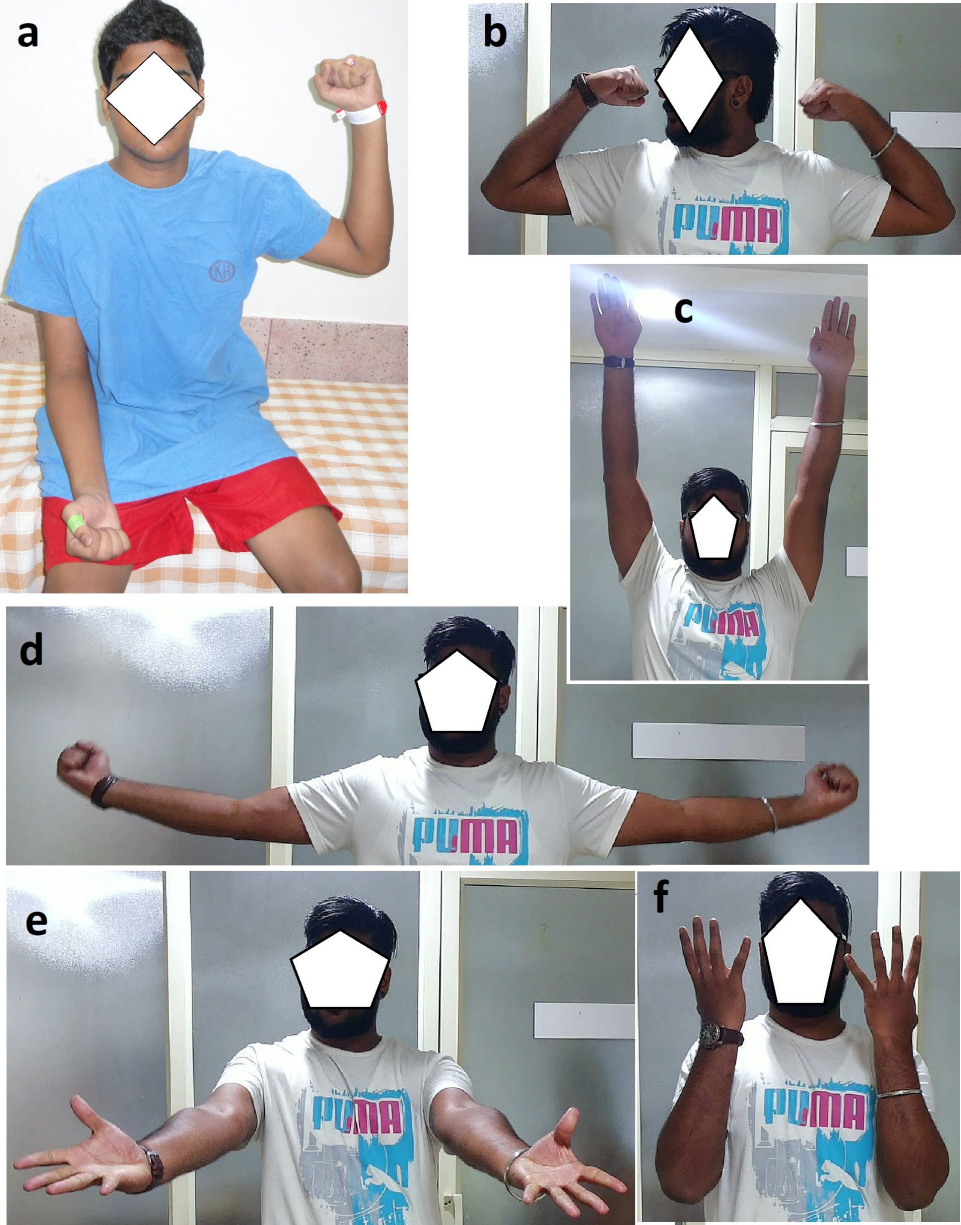
**a** Showing patient 8 who presented with upper brachial plexus injury with C5, C6 root level involvement. Underwent nerve transfer for suprascapular, axillary and Oberlin II for musculocutaneous nerves. **b**, **c**, **d** At 98 months follow up – showing M5 normal function of shoulder and elbow.**e, f** Patient could easily dissociate elbow flexion and forearm supination from wrist and hand movements

**Fig. 3:**
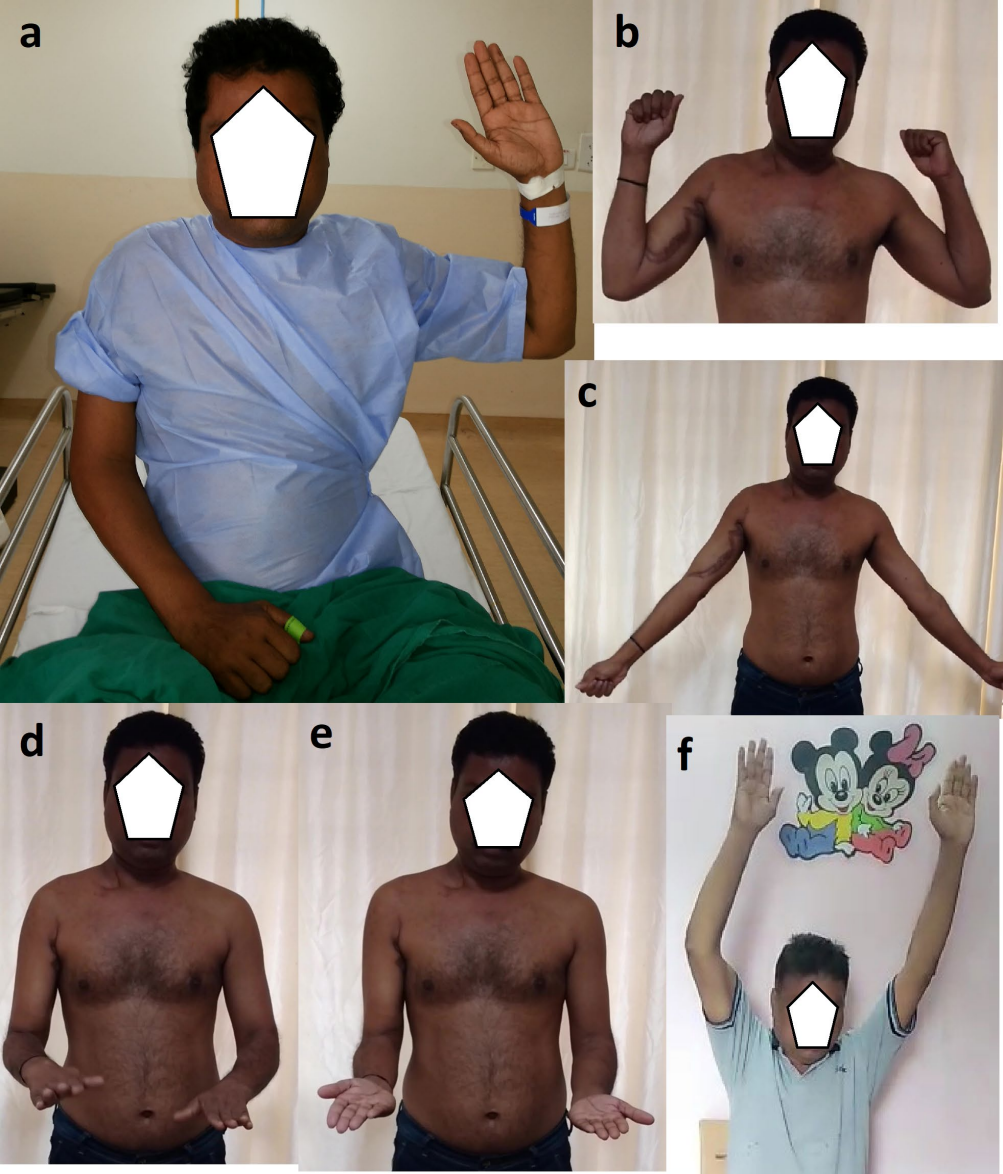
**a** Showing patient 16 who presented with C5, C6 root level injury. He underwent the same nerve transfer as shown for patient 8. **b**–**f** Showing the restoration of shoulder and elbow function at 74 months after surgery. Notice the independence of shoulder and elbow movements from wrist and hand movements in **d** and **e**

The study consisted of 16 men (89%) and two women with a mean age of 30 years (range—18–46 years). 13 patients (72.2%) had C5–C6 root/ upper trunk injury while five patients (27.8%) had involvement of C5-C7 roots/ upper and middle trunk (Table 1). The average time elapsed from the time of injury to surgery was 4.9 months (range: 2.5—9 months) (Table 1). Surgical delay did not influence the recovery of flexion or supination at any of the follow up timelines (*p* = 0.644 and 0.864 respectively at 5-year follow-up). None of the patients had any features of donor deficits at final follow-up. Pre-operatively, the MRC power grade for elbow flexion and supination was zero in all patients. 16 (88.9%) patients recovered to achieve active elbow flexion and forearm supination of either M3 power or more (p < 0.000) (Table 2). Based on Friedman’s two-way analysis of variance rank the recovery of flexion and supination were significant across both the categories of C5-6 root (*p* < 0.000 for both supination and flexion), and C5-7 root level injuries (*p* = 0.003 and 0.004 for supination and flexion respectively)0.14 (77.8%) of them achieved M4 or more for elbow flexion, with one achieving M5 power. 12 patients (66.7%) achieved M4 or more for forearm supination. Patients who achieved M3 or higher flexion also regained supination (Table 2). Two patients (case No.3 and 10) failed to achieve effective elbow and forearm function. Incidentally, both of those patients had a history of smoking.Nine patients (50%) achieved M3 active elbow flexion by the time of one year following surgery. At the end of two years, all the patients except two achieved the same or above. The recovery of supination was observed to follow the improvement in flexion. None of our patients could demonstrate supination against gravity at one year of follow-up. However, this had improved at two years with 11 patients (61.1%) showing M3 power or more (Table 2). The recovery continues even after two years of surgery as we observed five patients improved their MRC grade by one for elbow flexion and 15 patients for forearm supination.The average range of active elbow flexion movement was 113.9^°^ (range—0–140^°^) with those having grade 3 or more achieving an average of 128^°^. The average range of active supination was 67.8^°^ (0–90^°^). This reached a near-normal average of 76.3^°^ for patients who reported grade 3 power or more (Table 2) (Fig. 2). The distribution of recovery of ROM of flexion and supination was similar for both the C5-6 and C5-7 groups at the time points of two and 5 year follow-up (p = 0.775 and 0.173) for flexion and supination respectively.The mean QuickDASH score was 21.8 (range 2.3–63.6) (Table 1). The QuickDASH score showed a strong correlation with range of movement of flexion and supination based on Pearson’s R Spearman Correlation There’s a significant inverse relationship between Quick DASH with both flexion and supination at both 2- and 5-years measures. Both Pearson’s R and Spearman’s correlation correlations were statistically significant at p < 0.001 and < 0.05 respectively. We also observed that 13 (72.2%) of 18 patients were using the hand to eat without any aid or instrument. Patient having M3 power and less failed to achieve hand to mouth as the range of active movements at elbow and forearm was inadequate. One of the patients (case No.5) was unable to do due to multiple malunited metacarpal fractures. There was a difference in the distribution of abilities between the two injury levels, with C5-6 injuries generally showing better results. However this was not statistically (p = 0.101 > 0.05) significant. 15 (83.3%) patients could demonstrate independent finger and wrist flexion and extension along with pronation and supination of the forearm while steadily holding the elbow in 90^°^ flexion (Table 1) (Fig. 3). Two of them were unable to do this activity as there was no recovery of elbow flexion and in the other (case No.7) there was an associated wrist drop. The Pearson Chi-Square and Linear-by-Linear association tests did not show a significant association with the level of injury at the conventional 0.05 level (p = 0.099 > 0.05) although patients with C5-6 showed a trend towards better performance.

**Table 1:** Patient details with QuickDASH scores and two other activity important activity after 5 year follow up

No	Age	Sex	Level of injury	Surgical delay	Follow up period (months)	Quick DASH	Ability to eat with hand	Independence of elbow flexion and forearm supination from wrist and digital movements
1	32	M	C5,C6	3 months	74	9.1	Present	Present
2	42	M	C5,C6	3 months	75	27.3	Present	Present
3	22	M	C5,C6	5 months	77	50.0	Absent	Absent
4	38	M	C5,C6	3 months	85	20.5	Present	Present
5	21	F	C5,C6,C7	8 months	63	25.0	Difficult	Present
6	29	M	C5,C6	5 months	66	25.0	Present	Present
7	25	F	C5,C6,C7	5 months	61	34.1	Absent	Absent
8	21	M	C5,C6	2 months	98	2.3	Present	Present
9	22	M	C5,C6,C7	2.5 months	97	9.1	Present	Present
10	18	M	C5,C6,C7	3.5 months	98	63.6	Absent	Absent
11	21	M	C5,C6	9 months	88	6.8	Present	Present
12	33	M	C5,C6	4 months	64	23	Present	Present
13	26	M	C5,C6	3 months	96	24	Absent	Present
14	46	M	C5,C6,C7	6 months	89	11	Present	Present
15	23	M	C5,C6	3 months	63	5.5	Present	Present
16	46	M	C5,C6	9 months	74	19	present	present
17	26	M	C5,C6	6 months	86	20.5	Present	Present
18	45	M	C5,C6	8 months	75	15.9	Present	Present

**Table 2:** Showing the serial follow up assessment for MRC grading and range of movement for elbow flexion and supination of forearm

Patient No	3 months		6 months		12 months		2 years		After 5 years			
	F	S	F	S	F	S	F	S	F	F(ROM)	S	S(ROM)
**1**	0	0	1	0	3	2	4	3	4	0–130	4	0–90
**2**	0	0	1	0	3	0	4	3	4	0–140	3	0–70
**3**	0	0	0	0	0	0	1	0	1	0	0	0
**4**	0	0	1	0	3	2	4	3	4	0–120	4	0–70
**5**	0	0	0	0	2	0	3	2	4	0–130	3	0–60
**6**	0	0	0	0	3	2	4	3	4	0–130	4	0–70
**7**	0	0	0	0	2	0	3	2	3	0–90	3	0–60
**8**	1	0	2	0	3	2	4	4	5	0–135	5	0–90
**9**	0	0	1	0	2	0	4	3	4	0–140	4	0–80
**10**	0	0	0	0	0	0	0	0	0	0	0	0
**11**	1	0	1	0	3	2	4	3	4	0–130	4	0–80
**12**	0	0	1	0	3	0	4	3	4	0–120	4	0–60
**13**	0	0	1	0	2	0	3	2	3	0–110	3	0–90
**14**	0	0	0	0	2	0	3	2	4	0–135	4	0–80
**15**	1	0	1	0	3	2	4	3	4	0–140	4	0–80
**16**	0	0	1	0	2	0	3	2	4	0–140	4	0–80
**17**	0	0	1	0	3	2	4	3	4	0–130	4	0–70
**18**	0	0	0	0	2	0	3	3	4	0–130	4	0–90

F – flexion, S – supination, ROM – range of movement

## Discussion

Oberlin II procedure is currently a well-accepted procedure for reconstruction of elbow flexion in brachial plexus injuries as shown in systematic reviews and meta-analyses (Table 3) [4, 5]. However, we were unable to find any study with a follow-up of more than five years involving the Oberlin II procedure. There is also no detailed information on the return of supination following this procedure in the adult population. The current study addresses both issues and confirms the usefulness and reliability of the Oberlin II procedure demonstrating satisfactory return of function in the majority of our patients.In the only report involving greater than five years follow up six patients, Nagano et al. did partial ulnar nerve transfer to the biceps branch of MCN. They observed that five of their patients, maintained a minimum of M4 power at final evaluation for elbow flexion [7]. However, one of their patients incidentally had undergone an additional procedure of Steindler’s flexorplasty. Three of their patients showed poor outcomes in QuickDASH scores which were due to secondary development of syringomyelia and ongoing median and radial nerve palsy. They reported continuing improvement in elbow flexion in two of their patients after early recovery of M4 power [7]. However, there was no mention of the status of forearm supination. Our study with an average follow-up of 79.4 months reconfirms the usefulness of this procedure with 88.9% of patients achieving elbow flexion and forearm supination against gravity. Among patients achieving MRC grade 4 power and above, the range of active movements of both flexion and supination achieved is close to normal. We observed improvement even after two years following surgery as observed for five patients for elbow flexion and particularly 15 patients with supination. This could perhaps be due to patients returning to their routine activity of daily living, occupation and improvement in pain relief and changes due to cortical plasticity [24, 25]. We also observed that supination recovery is delayed when compared to flexion by around six months (Table 2). This could be due to the surgical technique we followed. The median nerve fascicle was much closer to the target of entry to the brachialis muscle than the ulnar nerve fascicle to the biceps (Fig. 1). The biceps recovery is hence delayed by a few months which could be the reason why nine of our patients showed good recovery of elbow flexion without similar recovery of supination in the first year. The Oberlin II procedure has shown mixed results for PROM [12, 26–29] (Table 3). The overall DASH scores in these reports appear to be relatively high. The importance of PROM cannot be undermined as it reflects significantly on the patient’s activity of daily living and satisfaction with the surgery [7, 25]. A major reason in these cases could be due to the delay in surgery and a shorter follow up period [26–29].In our series, the QuickDASH scores were much better showing a strong statistical correlation reflecting the objective outcomes with those having M3 power performing better at more than five years follow up (Table 3). The average delay in surgery was less than five months and with our observation of continuing recovery even after two years, we believe that in the long run, results and function improved for reasons mentioned earlier. The two patients who failed to show motor recovery scored poorly with the QuickDASH scores (Table 1). A concern associated with this procedure is the presence of co-contracture of elbow flexors with the wrist and finger long flexors [9]. In a study involving 18 patients undergoing Oberlin’s procedure, Escudero et al. observed that 38.9% of their patients (group 1) were unable to dissociate elbow flexion from wrist and fingers flexion [15]. Although this group performed on Sollerman protocol similar to the second group who could dissociate the two sets of flexion, there was a significant difference in DASH scores in favor of group two which they attributed to the presence of co-contraction. However, this study involved surgery on the biceps branch alone and had a minimum of only one year follow-up. In another study which involved a follow-up study of around three years, Chia et al. compared the results of 23 ulnar nerve fascicle with 15 intercostal nerve transfers for elbow flexion in upper brachial plexus injuries [10]. They showed a significantly stronger eccentric contraction after ulnar nerve fascicle transfer using the evaluation of manual muscle testing, electromyography and dynamometry. Based on electromyography, they observed that all the patients with ulnar nerve fascicle transfers were unable to achieve isolated voluntary contraction of biceps and recruited both forearm flexors and extensors with significantly greater activation of the forearm muscles during concentric elbow flexion. However, only 17 patients out of 38 were recruited for electromyography. The time from surgery and the number of patient with Oberlin’s transfer for this assessment was also not mentioned and neither were PROM reported. In our opinion, a PROM at long-term follow-up would have added significant value and usefulness to this procedure. In this regard, we used the QuickDASH score which in our opinion has all the components to evaluate the patients’ ability to flex the elbow, supinate the forearm and more importantly assess the independence of this movement from those of the wrist and the digit. To assess independent supination further, we also assessed the patients’ ability to bring the hand to mouth for eating and to voluntarily demonstrate independent pronation and supinate forearm at 90^°^ elbow flexion with wrist and finger extended. We observed that independent movements were observed in almost all the patients with grade 3 power. The hand to mouth was influenced by the range of active movement of flexion and was absent in patients with grade 3 power and below. In one patient (Case 5) there were multiple malunited metacarpal fractures which prevented this activity. The patient did not agree for a corrective osteotomy later.Two of our patients showed no recovery of function. Incidentally, both had a history of smoking. One of them did undergo reexploration surgery at a different center but showed no signs of improvement. Both the patients were offered salvage surgery but refused. The limitation of our study is the retrospective nature of evaluation due to which serial information on objective and functional assessment on some of the patients could not be collected due to irregular follow-up. Given the limited number of patients a stronger statistical evaluation could not be assessed for which a multi-centre study would be valuable. Nevertheless, our study had a much larger volume of patients with a longer follow up providing new insights on this unique procedure.The Oberlin II procedure reliably restores elbow flexion and forearm supination which is independent of wrist and finger movements with satisfactory PROM in brachial plexus injuries in the long term.

**Table 3:** Reports of results of Oberlin I and II procedures which reported PROM along with the original technique article

Author	Y ear	No^*^	Age	Timing of Surgery (mths)^#^	Follow up (mths)	ROM^a^(Elbow) < 100			MRC^b^ (elbow) > 100	< 3 4/53	Supination MRC ≥ 3/ ROM ≥ 60^0^	PROM^c^ (score)
Original report												
Oberlin et al. [1]	1994	4	23	4.7	14	NA	NA	NA	100%	NA	NA	
Mackinnon et al. [2]	2005	6	34	4	20.5	NA	NA	NA	NA	NA	N/A	NA
Liverneaux et al. [3]	2006	10	27.2	6.6	15	NA	NA	NA	NA	100%	NA	NA
Oberlin I												
Escudero et al. [14]	2016	18	29.5	7	12	NA	NA	NA	NA	NA	NA	DASH- 51
Verdins et al. [7]	2018	10	38	11.7	44	NA	NA	NA	NA	100%	NA	DASH—27.3
Filho et al. [28]	2019	18	30	9.2	NA	22%	78%	22%	28%	50%	NA	DASH—37.9
Nagano et al. [6]	2019	6	29.5	5.3	156	NA	NA	16%	17%	67%	NA	QuickDASH—36.5
Oberlin II												
Nath et al. [27]	2006	40	25.2	5.2	NA	NA	NA	5%	5%	90%	N/A	WHO QOL^d^
Carlsen et al. [11]	2010	32	32	6	17	NA	NA	10%	10%	80%	Strength measured: 37% of contralateral side	DASH-28.6
Dolan et al. [25]	2011	9	31.4	6.6	24.8	33%	67%	22%	11%	67%	NA	DASH, SF-36, PVAS^e^ were done but not separately provided
Maricq et al. [26]	2014	3	29.3	4.3	25.3	NA	NA	0%	33%	67%	NA	DASH -60.8
Current Study	2021	18	30	4.9 m	79.4	17%	83%	11%	11%	77.8%	88.9%	QuickDASH—21.75

^*^ Number of patients, ^#^ Months, ^a^ Range of Movement, ^b^ Medical Research Council, ^c^ Patient reported outcome measure, ^d^ WHO QOL – World Health Organization – Quality of Life, ^e^ Pain Visual Analogue Score
